# *Salvia coccinea* and Apigenin: A Natural Treasure of Lamiaceae in Pharmacological Innovation

**DOI:** 10.1002/fsn3.71354

**Published:** 2026-02-03

**Authors:** Muhammad Usman Khalid, Muhammad Tauseef Sultan, Muhammad Maaz, Shehnshah Zafar, Anum Shoukat, Nudrat Khursheed, Matteo Bordiga, Amna Junaid

**Affiliations:** ^1^ Faculty of Food Science and Nutrition Bahauddin Zakariya University Multan Pakistan; ^2^ Department of Pharmaceutical Sciences Università Degli Studi del Piemonte Orientale “A. Avogadro” Novara Italy

**Keywords:** anticancer, apigenin, hypoglycemia, inflammatory cytokines, medicinal plants, *Salvia coccinea*

## Abstract

Human health and ever‐increasing disease burden demand the inclusion of traditional medicines in modern healthcare sectors. Phytochemicals extracted from medicinal plants can be utilized to prepare nutritious and quality food products that could offer nutritional and curative benefits. This review highlights the nutritional, phytochemical, and therapeutic profile of *Salvia coccinea* and its bioactive compound i.e., apigenin. *Salvia coccinea* , the urban green, scarlet, lance‐shaped flower, is cultivated in warm climatic conditions from summer to autumn. The micronutrient‐dense (sodium, calcium, potassium, zinc, nitrogen, and copper) leaves can prevent micronutrient‐deficiency disorders among consumers. Furthermore, apigenin along with other bioactive constituents e.g., luteolin, flavonoids, and phenolic acids offer strong antioxidants, anticancer, anti‐inflammatory, antidiabetic, antimicrobial, and anti‐cardiovascular properties. The free radical scavenging potential of *Salvia coccinea* and apigenin is responsible for reduced oxidative stress and tumor cell metastasis modulated through PARP‐cleavage, caspase‐3, ERK, CDK‐1, JAK2/STAT3, Bax/Bcl‐2, AMPK, and Wnt/β‐catenin pathways. They attenuate inflammation‐induced disorders such as cardiovascular and neurological disorders via down‐regulating pro‐inflammatory cytokines (IL‐6, CRP, COX‐2, LPO, TGF‐β1, NF‐κB, and TNF‐α), and pathways (IRAK4, MAPK, JAK/STAT3, TLR4, and ERK). The antimicrobial properties against multiple bacterial, viral, and fungal strains make them effective candidates for alleviating microbial disorders. Furthermore, apigenin and *Salvia coccinea* promote hypoglycemic effect by attenuating α‐amylase activity, cholesterol levels, insulin resistance, DRP1 expression by improving GLUT4, GSK‐3β, AMPK/PI3K/Nrf2, and Akt pathways. Moreover, *Salvia coccinea* regulates wound healing after infection, injury, or surgery, in addition to improving agricultural productivity by reducing rodent attacks.

## Graphical abstract

*Salvia coccinea* is rich in bioactive phytochemicals, notably the flavonoid apigenin, offering strong antioxidant, anti‐inflammatory, and antimicrobial effects. By modulating inflammatory and oxidative pathways, it shows potential for managing chronic diseases. This supports its use in developing novel pharmaceutical and nutraceutical applications.

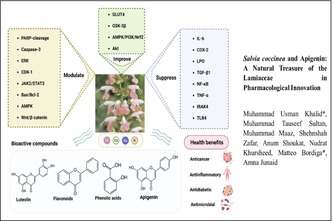

## 1 Introduction

**FIGURE 1:**
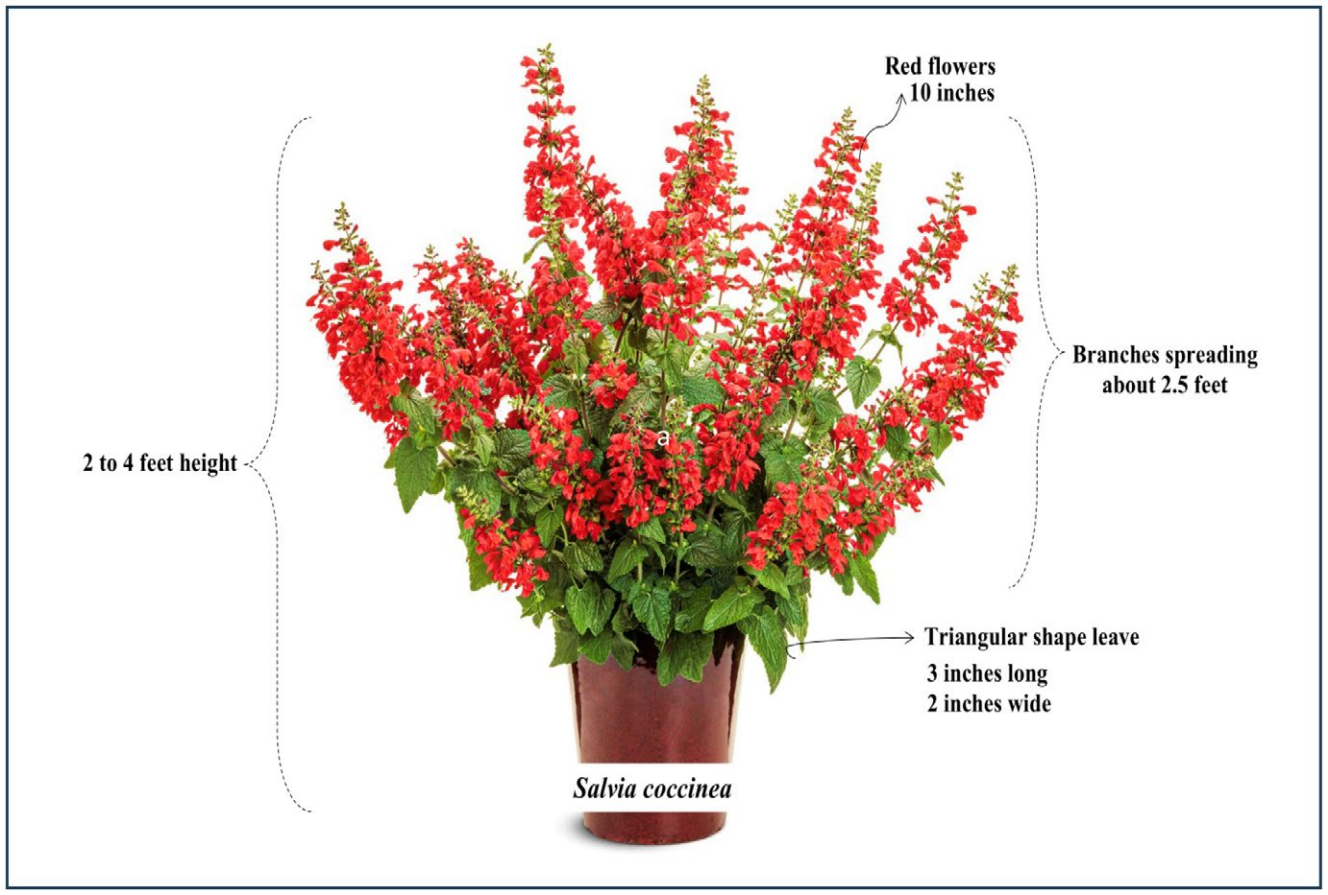
Botanical description of *Salvia coccinea*.

Medicinal and herbal plants have gained importance in the healthcare sectors for the last few years. The increased disease prevalence and harmful impacts of medications encourage scientists and healthcare professionals to use medicinal plants to improve individuals' health (Dahiya and Garg 2024). Humans are rewarded with diverse medicinal plants, such as *Curcumin longa*, *Citrullus colocynthis* , *Thymus vulgaris* , *Prunus domistica*, *Withania somnifera* , *Emblica officinalis, Saraca Asoca*, *Phyllanthus amarus* , which constitute diverse bioactive compounds, such as flavonoids, flavonols, polyphenols, anthocyanins, carotenoids, and saponins (Maaz et al. 2025; Lambo et al. 2024). Moreover, functional foods and nutraceuticals are prepared using bioactive compounds that modulate normal health and well‐being (Mudondo et al. 2025). These products reveal antioxidant, anticancer, anti‐inflammatory, antimicrobial, hepatoprotective, cardioprotective, and neuroprotective properties, reducing the prevalence of metabolic and cardiovascular disorders (Ansari et al. 2025). *Salvia coccinea* (Lamiaceae), a medicinal plant belonging to the *Lamiaceae* family, is renowned as banderilla, blood, tropical, Texas, or crimson sage. It is an esteemed species of sage throughout the southeast United States, Mexico, the Caribbean, North America, South Africa, and the eastern Himalayas (Deshmukh 2022). It is widely grown in urban green and private gardens of the world, having long flowering durations from summer to autumn. It has maximum growth in warm climatic conditions. *S. coccinea* is 2–4 ft tall, with its branches spreading about 2.5 ft. Its leaves are hairy, triangular, pea green in color, and have varying sizes, i.e., 3 in. long and 2 in. wide. It has a variable flower size (10 in. long) and color. The nectar produced by flowers captivates hummingbirds and butterflies, aiding pollination (Karalija et al. 2022). The botanical description of *S. coccinea* was illustrated in Figure 1. It has been traditionally used to cure ulcerative colitis, pneumonia, flatulence, anorexia, and nausea (Ghoran et al. 2023). Its floral parts are used in Mexico to treat blood clots and congestive heart failure in Mexico and South America. Moreover, the emmenagogue, carminative, antiemetic, diaphoretic, antioxidant, antifungal, sedative, anti‐inflammatory, antispasmodic, and anti‐catarrhal properties of *S. coccinea* alleviate the disease burden of society (Zambare et al. 2021). This review highlights the botanical, geographical, nutritional, phytochemical, and pharmacological characteristics of *S. coccinea* . Moreover, the rodent, pre‐clinical, and experimental trials regarding the health benefits of *S. coccinea* and its bioactive constituent, apigenin, are comprehensively described alongside their industrial applications.

## 2 Methodology

This literature review was conducted to identify, analyze, and synthesize existing research on *S. coccinea* and its bioactive constituent, apigenin. Data was assembled using databases such as PubMed, ScienceDirect, Google Scholar, Scopus, Web of Science, and Directory of Open Access Journals, mainly from the last 10 years. Moreover, data was searched by utilizing different keywords and Boolean operations (and & or), e.g., medicinal plants, *Salvia coccinea* , phytochemicals, nutritional profile/composition, antioxidant and *Salvia coccinea* , antioxidant and apigenin, anticancer and *Salvia coccinea* , antioxidant and apigenin, anti‐inflammatory and *Salvia coccinea* , anti‐inflammatory and apigenin, antimicrobial/antibacterial/anti‐fungal and *Salvia coccinea*, antimicrobial and apigenin, cardioprotective/neuroprotective and *Salvia coccinea*, cardioprotective, neuroprotective and apigenin, wound healing/skin health and *Salvia coccinea* , and industrial application of *Salvia coccinea* . The in vitro, in vivo, pre‐clinical, clinical, and rodent studies were included in this review; however, studies other than the last 10 years, non‐English language, and restricted journals were excluded to make the review effective, readable, and understandable. Table 1 shows the taxonomy classification of *Salvia coccinea* .

**TABLE 1:** Taxonomical classification.

Kingdom	*Plantae*
Clade	*Tracheophytes*
Clade	*Angiosperms*
Clade	*Eudicots*
Clade	*Asterids*
Order	*Lamiales*
Family	*Lamiaceae*
Genus	*Salvia*
Species	*Salvia coccinea*

## 3 Nutritional and Phytochemical Composition

The nutritional profile of plants is significant in determining the extent of consumers' acceptability. Plants with poor nutritional characterization led to the development of nutrient deficiency disorders. Factors such as soil fertility, irrigating water quality, environmental contaminants, and seasonal fluctuations influence the nutritional profile and overall acceptability (Usowicz and Lipiec 2021; Walling and Vaneeckhaute 2020). Studies were conducted to determine the nutrient contents of *S. coccinea* , which found that its leaves constitute an abundant amount of minerals, such as manganese (97.4%–149%), zinc (52.30%–64.30%), boron (27.7%–31.4%), nitrogen (3.98%–5.99%), potassium (1.71%–2.98%), calcium (1.23%–1.75%), phosphorous (0.46%–0.64%), molybdenum (0.24%–0.36%), magnesium (0.27%–0.66%), copper (2.66%–2.99%), and sodium (0.04%–0.09%) (Sehnal et al. 2019; Salachna et al. 2019).

**FIGURE 2:**
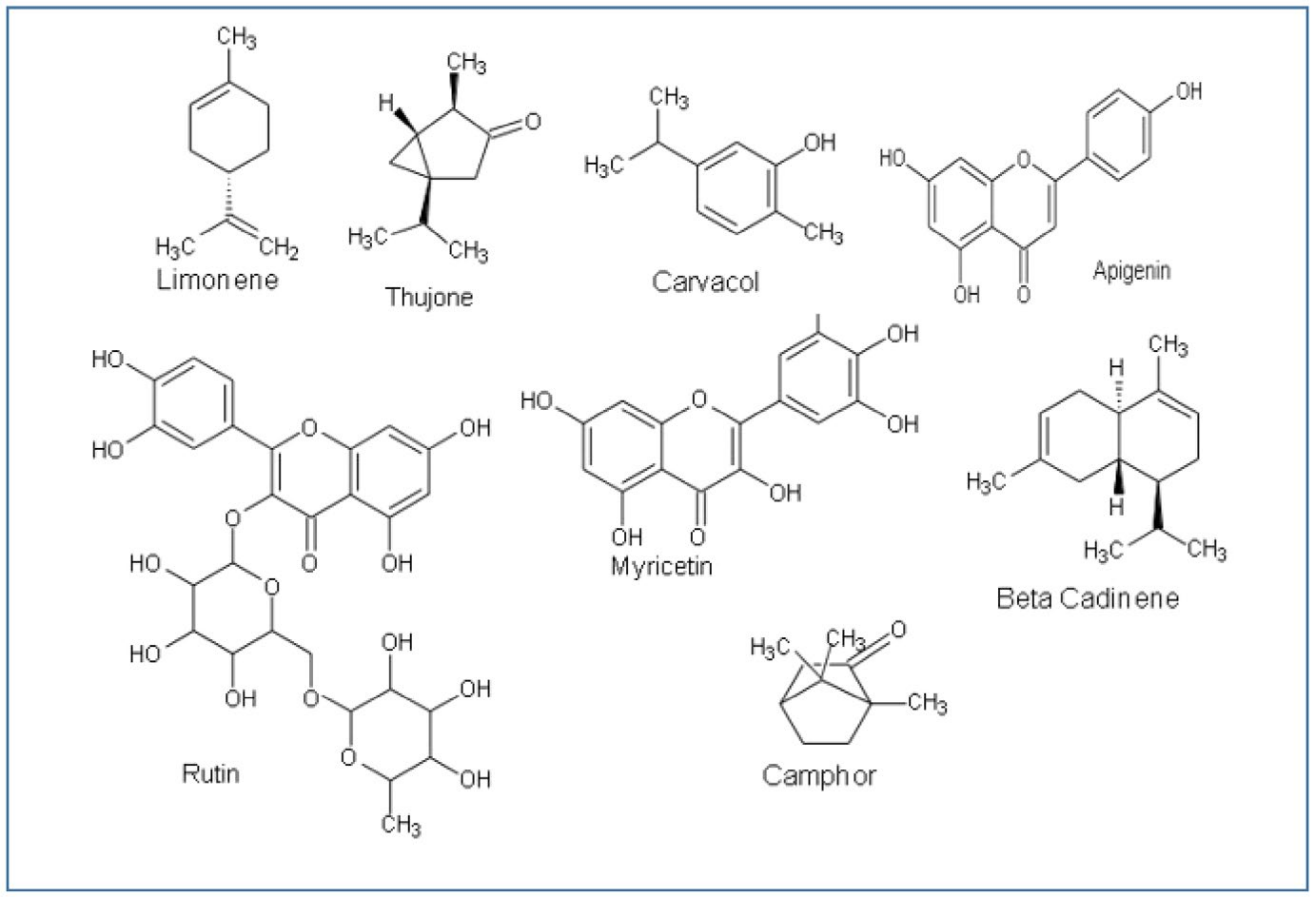
Phytochemical constituents of *Savia coccinea*.

Phytochemicals are the bioactive compounds found in herbs, plants, fruits, and vegetables, which interact with other compounds to strengthen the immune system and ultimately alleviate the pathogenesis of disorders. It has been declared that *S. coccinea* consists of bioactive components (depicted in Figure 2), such as flavonoids, phenols (coumaric, chlorogenic, sinapic, and ferulic acid), terpenes, and alkaloids, which are responsible for unveiling pharmacological attributes (Agatonovic‐Kustrin et al. 2023; Sabry et al. 2022; Li, Yang, et al. 2022; Li, Wu, et al. 2022; Zúñiga‐López et al. 2021; Miraj and Kiani 2016). A study was conducted to investigate the chemical characterization of methanolic and petroleum ether‐prepared *S. coccinea* oleoresins through GC–MS analysis. It has been revealed that oleoresins contain oleic acid, palmitic acid, squalene, limonene, β‐Sitosterol, stigmasterol acetate, stigmasta‐3,5‐dien‐7‐one, phytol, neophytadiene, and phthalic acid (Nagarkoti et al. 2023). Moreover, camphor, α‐ and β‐thujone, viridiflorol, α‐humulene, cineol, manool, n‐hexadecanoic acid, γ‐amorphene, beta‐caryophyllene, dimethoxy cymene, germacrene, sesquiterpene hydrocarbons, α‐globulol, aromadendrene, 2‐trans hexane, and acenaphthene have been isolated from *S. coccinea* (Aslani et al. 2023; Rajendran et al. 2022; Mohan et al. 2019). Other compounds identified from the *S. coccinea* are thujone (Ezema et al. 2022), apigenin (Sabry et al. 2022), 1,8‐cineole (Ehrnhöfer‐Ressler et al. 2013), 7‐hydroxyapiana dieneolide (Hafez Ghoran et al. 2022), saprorthoquinone (Dziadek et al. 2022), rutin (Remigante et al. 2022), Salvisyriacolide (El Euch et al. 2019), salvianolic acid (Mathew and Thoppil 2011), β‐cadinene (Venkatesan et al. 2023), β‐pinene (Ninkuu et al. 2021), α‐santonin (Kandikattu and Singh 2013), myricetin (Cao et al. 2023), tetrandrine (Dinis‐Oliveira et al. 2019), triptolide (Agatonovic‐Kustrin et al. 2023), carvacrol (Sampaio et al. 2021), chalcones (Teles et al. 2018), and dihydroflavonol (Kumar and Patil 2020). These compounds promote the health‐promoting and disease‐reducing properties of *S. coccinea* .

## 4 Medicinal Properties of *Salvia coccinea* and Apigenin

*S. coccinea* exhibits diverse pharmacological attributes, such as anti‐inflammation, anti‐oxidative, anti‐proliferative, anti‐diabetic, and hepatoprotective, due to the presence of bioactive compounds in the apical portion. Moreover, health benefits associated with its bioactive compound, apigenin, have also been comprehensively described.

## 5 Antioxidant Potential

Antioxidants are the free radical scavengers that modulate normal health by reducing the pathogenesis of disorders such as type 2 diabetes mellitus, hyperlipidemia, neurodegenerative diseases, atherosclerosis, and arthritis. These attributes encourage food scientists, industrialists, and consumers to utilize medicinal plants for the betterment of health and to meet the needs of growing populations. Several studies have been conducted to determine the antioxidant potential of *S. coccinea* , and their details are described here in detail. The free radical scavenging activity of plants and compounds is affected by the hydrogen‐donating ability, position, and the extent of hydroxylation (Pápay et al. 2017). The antioxidant potential of ethanol (50% and 100%) and methanol (100%) extract of *S. coccinea* was determined by conducting a DPPH assay. The extracts from 50% and 100% ethanol and 100% methanol solution revealed the maximum antioxidant potential. However, the lowered antioxidant capacity of residues was observed from pure water and 25% ethanol treatment (Gouda et al. 2021). Previously, the antioxidant potential of methanol leaf extract of *S. coccinea* and *S. officinalis* was evaluated by conducting superoxide, nitric oxide, hydroxyl, and DPPH radical scavenging activities. It was observed that the mean IC50 values of *S. officinalis* and *S. coccinea* were found to be 7.34 and 8.79 mg/mL, respectively (Yadav and Mukundan 2011). Different factors, particularly salt stress and salicylic acid, affect the chemical and phytochemical composition of *S. coccinea* . It has been revealed that the total polyphenol contents of *S. coccinea* leaves were significantly reduced in the NaCl group (45 mg GAE/g DM) as compared to the normal control group (78 mg GAE/g DM). Moreover, a higher antioxidant profile (1461.56 mg TE/g DM) assessed by FRAP assay was observed in *S. coccinea* leaves treated with 200 mM NaCl and 0.5 mM salicylic acid (Grzeszczuk et al. 2018). The antioxidant activity of apigenin, a bioactive compound of *S. coccinea* , is confirmed by Kim et al. (2019) and Sadasivam and Kumaresan (2011). Furthermore, the antioxidant potential of apigenin was evaluated by conducting ABTS and FRAP assays, and their respective IC50 values were found to be 0.82 μg/mL and 0.01 mmol Fe2+/mg/mL. Moreover, the DPPH assay was used to determine the antioxidant activity of apigenin‐loaded bovine serum albumin nanoparticles, and it confirmed the free radical scavenging capability of encapsulated apigenin (Pápay et al. 2017).

## 6 Anticancer Property

Cancer, one of the significant causes of mortality worldwide, has disturbed the quality of life of individuals by compromising immune response. Cancers, such as breast, esophageal, pancreatic, gastric, colorectal, brain, bone, and cervical, are prevailing among communities due to unhealthy eating practices, sedentary lifestyles, contaminated environmental conditions, and climatic modifications. Chemotherapeutic drugs are commercially available to attenuate the metastasis of cancerous cells. However, these drugs contribute to the pathogenesis of other maladies, such as cognitive impairment, renal injury, anemia, and gastrointestinal disturbances. Medicinal and herbal plants have been employed alone or in combination with chemotherapeutic drugs and compounds to alleviate the adverse consequences of these drugs (Noman et al. 2025). *S. coccinea* , a medicinal plant, has been shown to exhibit cytotoxic properties, as evidenced by several studies. Rajendran et al. (2023) prepared silver nanoparticles of *S. leucantha*, *S. splendens* , and *S. coccinea* and investigated their potential against human lung cancer cells (A549). The results have shown that their silver nanoparticles have anticancer properties with the respective IC50 values of 364, 418, and 402 μg/mL. Moreover, the antiproliferative potential of zinc oxide nanoparticles of *S. coccinea* leaf extracts was shown against MCF‐7 and A431 cell lines. It has been revealed that these nanoparticles reduced cancer proliferation by 68% and 64%, respectively (Kirubakaran et al. 2024). Previously, apigenin demonstrated anticancer potential by regulating immune response and reducing cancer cell proliferation (Xu et al. 2021). The effect of apigenin against the metastasis of liver cancer cell lines, such as PLC and Bel‐7402, was determined. It was found that apigenin reduced their proliferation, migration, and invasion by decreasing Snai 1, NF‐κB, and epithelial‐mesenchymal transition (EMT), thereby showing a protective effect of apigenin (Qin et al. 2016). Similarly, apigenin alleviated the progression of HepG2 cell lines by elevating PARP‐cleavage, caspases, and ERK pathways (Kim et al. 2013). Furthermore, the synergistic administration of apigenin with other chemotherapeutic drugs, such as 5‐Fluorouracil, cetuximab, cisplatin, cyclophosphamide, doxorubicin, paclitaxel, and tamoxifen, has improved cancer patients' survival by downregulating tumor cell proliferation, mitochondrial membrane potential, and by increasing total antioxidant capacity (Nozhat et al. 2021). Further studies regarding the antimetastatic potential of apigenin are presented in Table 2.

**TABLE 2:** Anticancer mechanism of apigenin against the proliferation of cancers.

Cancer types	Cell lines	Mechanism	References
Breast cancer	MDA‐MB‐231, MDA‐MB‐453, and T47D	↓cyclin‐dependent kinase‐1 (CDK‐1) activity, ↓STAT3 pathway, ↓JAK2 and STAT3 phosphorylation, ↑caspase‐3, PARP cleavage, and Bax/Bax‐2 activity	Tseng et al. (2017), Noolu et al. (2016), and Seo et al. (2014)
	MDA‐MD‐468 and 4 T1	↑immune functionality by modulating IFN‐γ, programmed death ligand 1 (PD‐L1), and STAT 1	Coombs et al. (2016)
	TNBCs, MCF‐7, JIMT‐1, and MDA‐MB‐436	↓cell proliferation, invasion, and migration of tumor cells, ↓YAP/TAZ activity, ↓CTGF, ↓SIRT3 and SIRT6, ↓TNF‐α and NF‐κB	Sharma et al. (2022), Tuasha et al. (2022), and Li et al. (2018)
Thyroid cancer	Human 8505C, CAL62 ATC, and BCPAP	↑Bcl2, ↑autophagy, ↑DNA damage	Kim et al. (2015) and Zhang et al. (2015)
Colorectal cancer	SW480 cells, DLD‐1, LS174T, and HCT116	↓proliferation, ↓Wnt/β‐catenin, ↑FADD and TAGLN expression, ↓MMP‐9, ↓cyclin B1, ↑p53, and ↑PARP cleavage	Xu et al. (2016), Lee et al. (2014), Chunhua et al. (2013), and Wang et al. (2011)
	HT‐29 cell lines	↓cancer cell proliferation and metastasis with the IC_50_ value of 2.03 μM	Zheng et al. (2014)
Prostate cancer	PC‐3, 22Rv1, and DU145	↓E‐cadherin/snail expression, ↓IKKα kinase activity, ↑apoptosis, ↓NF‐κB, ↓oxidative DNA damage, ↓proteosome chymotrypsin‐like activity, ↓HDACs class 1, and ↑Apo2L/TRAIL‐induced apoptosis	Singh et al. (2015), Zhu et al. (2015), Shukla et al. (2014), and Pandey et al. (2012)
Cervical cancer	Hela	Inhibit cell proliferation by targeting CK2α	Liu, Cao, et al. (2015)
	Hela and C33A	↓cell viability, ↓cell cycle progression at G0/G1 and S phase, CDk1, cyclin B1, and CDC25c	Chen et al. (2022)
Osteosarcoma	U2OS and MG63	Downregulated tumor cell proliferation, invasion, and metastasis by affecting Wnt/β‐catenin expression	Liu, Li, et al. (2015)
Ovarian cancer	A2780 and SKOV3	↓Tumor growth, invasion, and metastasis by regulating FAK and CK2α gene expression	Tang et al. (2015) and Hu et al. (2008)
Brain cancer	U87MG and U373MG	↓CD133, ↓NANOG, ↓c‐Met, ↓Akt, and ↓SOX2	Kim et al. (2016)
Hepatic carcinoma	SK‐Hep‐1	↓Bcl‐2, ↑Bax, ↑miR‐34a, ↓NANOG, ↓ALDH1 and ABCG2	Xu et al. (2020)
	HCSLCs	↓MnSOD, ↓FoxM1, ↑apoptotic pathway, ↓tumor cell proliferation	Cao et al. (2019)
Lung cancer	A549, H1299, and LCSLCs	↑p53, ↓MnSOD/CaMKII/AMPK pathway, ↓Bcl‐2, ↓NF‐κB, ↓ERK, ↑Bad and Bax expression	Li, Zhao, et al. (2021), Li, Chen, et al. (2021), Liu et al. (2021), Chen et al. (2016)
	A549 cells	↓depolymerized microtubules, ↑apoptosis, ↑cell death, IC50 = 79 μM	Choudhury et al. (2013)
	A549 and H1299 cell lines	↑DR4 and DR5, ↑Bax and Bad, ↓Bcl‐xL and Bcl‐2	Cardenas et al. (2016)
	H460 cell lines	↓cell viability, ↑apoptosis and DNA damage, ↓Bcl‐2 and Bid, ↑Bax	Lu et al. (2011)
Leucocythemia	HL‐60 cell lines	Attenuated cancer propagation and invasion with the IC_50_ value of 2.25 μM	Zheng et al. (2014)
Melanoma	A375 and C8161	↓proliferation and invasion, ↑ cleaved caspase‐3 and PARP, ↓p‐AKT, ↑p‐mTOR, ↓ERK1/2 protein	Zhao et al. (2017)
	A2058, A375, and G361	↓STAT3, MMP‐2, MMP‐9, and VEGF, and ↓FAK/ERK1/2	Cao et al. (2016) and Hasnat et al. (2015)

Inflammation is the physiological response of immune cells in case of a foreign pathogen's entrance. Inflammatory cytokines promote endothelial dysfunction that attracts monocytes into the arterial wall. Monocytes form foam cells by differentiating into macrophages and oxidized low‐density lipoprotein, ultimately producing proinflammatory markers, such as interleukin‐6 (IL‐6), C‐reactive protein (CRP), and matrix metalloproteinases (MMP), to reveal an inflammatory response. Prolonged and uncontrolled inflammation leads to inflammatory disorders, such as certain metabolic and cardiovascular disorders (Attiq et al. 2024). Medicinal plants, particularly *S. coccinea* , hold anti‐inflammatory potential, as evidenced by various studies. For instance, the biogenic silver nanoparticles (ScAgNPs) were produced from *S. coccinea* leaves, and their anti‐inflammatory response was evaluated in monocytic THP‐1 cells. The results depicted that ScAgNP administration (100–400 μg/mL) reduced glucose‐induced oxidative stress and prevented NF‐κB‐driven transcription of proinflammatory genes in THP‐1 cells (Shanmugam et al. 2021). Similarly, carrageenan‐induced pleural edema was reduced to 40.7% by the oral administration of hydro‐alcoholic extract of *S. coccinea* leaves. Moreover, the aqueous extract of *S. coccinea* leaves inhibits NF‐κB and scavenges free radicals, demonstrating their anti‐inflammatory properties in diabetic rats (Sudaramoorthy et al. 2021).

Apigenin, the bioactive compound found in *S. coccinea* , exhibits anti‐inflammatory properties by suppressing interleukin‐1 receptor‐associated kinase 4 (IRAK4), NF‐κB, and MAPK pathway, thereby attenuating the prevalence of chronic inflammatory disorders (Gongpan et al. 2025; Ginwala et al. 2019). Recombinant tissue plasminogen activator is an effective strategy for the management of ischemic stroke, but it negatively affects the blood–brain barrier and even causes a patient's demise. Apigenin has a protective effect on blood–brain barrier integrity, and its exact mechanism was investigated by Wang, Yan, et al. (2024), Wang, Yu, et al. (2024). It has been revealed that the anti‐inflammatory potential of apigenin improved blood–brain barrier integrity by targeting MMP‐9, Jak/STAT3, and TLR4 pathways. In another study, the effect of apigenin on indomethacin‐induced gastric ulcers was investigated. It was concluded that apigenin reduced gastric ulcers by reducing lipid peroxidation (LPO), cyclo‐oxygenase‐2 (COX‐2), NF‐κB, and TNF‐α, as well as by improving transforming growth factor‐beta 1 (TGF‐β1), SOD, and CAT (Alamri 2024).

Furthermore, ocular inflammation has an adverse impact on the physiology of the eye, leading to the occurrence of vascular and neurodegenerative disorders. Apigenin has profound anti‐inflammatory activity; however, apigenin therapy in combination with melatonin significantly attenuates ocular inflammation. Bonilla‐Vidal et al. (2025) revealed that apigenin and melatonin synergistically reduced ocular inflammation by alleviating interleukin‐6 (IL‐6), interleukin‐8 (IL‐8), and monocyte chemoattractant protein 1 (MCP‐1) levels. Similarly, the synergistic anti‐inflammatory effect of apigenin and genistein was evaluated in rat intestinal epithelial cells. It was observed that the production of prostaglandin E2, IL‐6, IL‐1β, NF‐κB, and TNF‐α was significantly decreased by apigenin and genistein treatment. Moreover, apigenin and genistein improved IL‐10 (anti‐inflammatory cytokine) and transforming growth factor‐β (TGF‐β) levels (Cai et al. 2022). Previously, the anti‐inflammatory potential of apigenin was assessed among Wistar rats with acute pancreatitis, and their effect was observed at different time intervals, i.e., 6, 12, 24, 48, and 72 h. The results depicted that apigenin lowered pancreatic necrosis by downregulating TNF‐α expression (Charalabopoulos et al. 2019).

**FIGURE 3:**
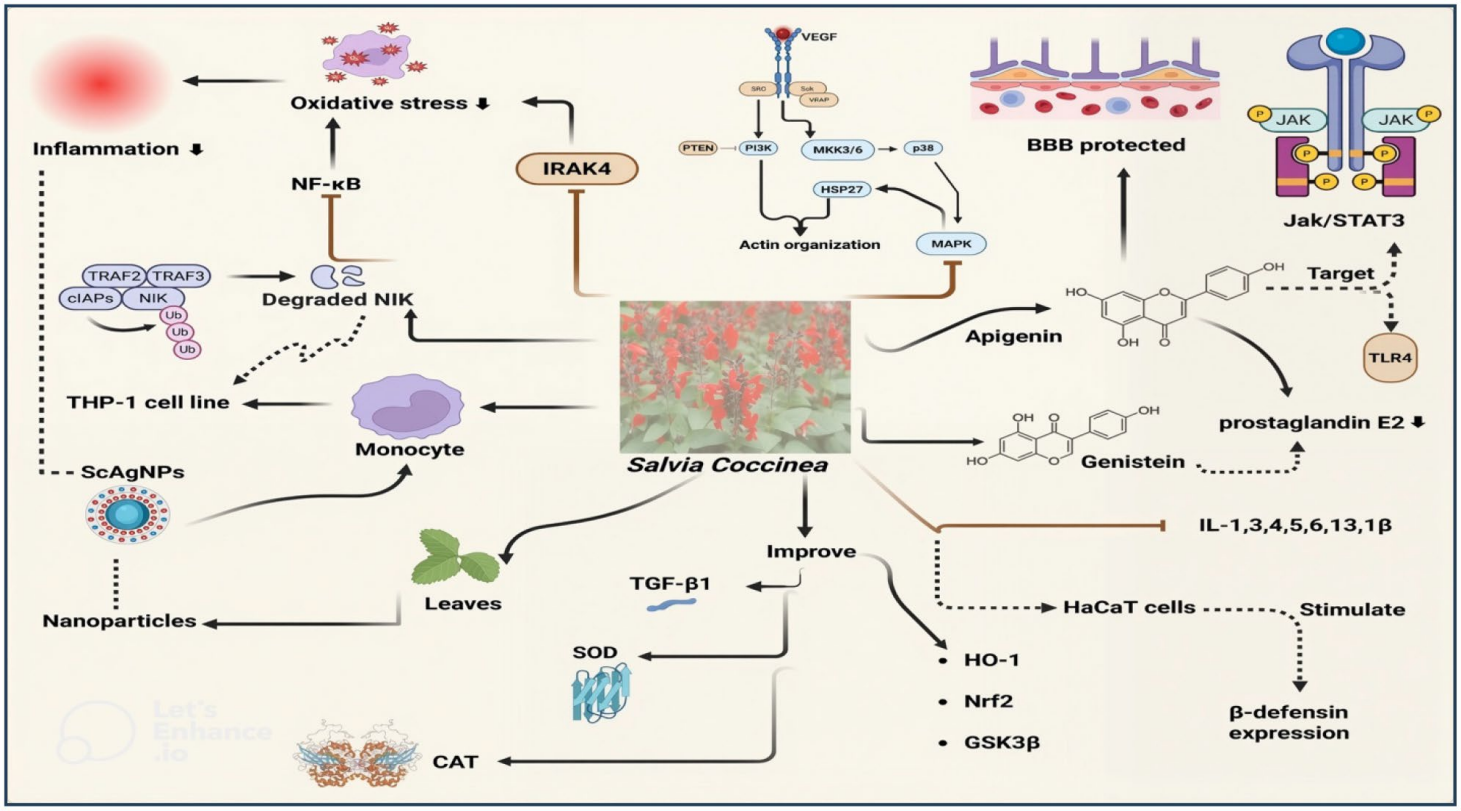
Anti‐inflammatory potential of *Salvia coccinea* and apigenin.

The inflammatory and allergic‐producing pathways are also inhibited by apigenin administration. It has been disclosed that nitric oxide, IL‐4, IL‐5, IL‐6, IL‐13, IL‐1β, TNF‐α, iNOS, COX‐2, MAPK, Lyn, Syk, JNk, and ERK pathways are inhibited by apigenin supplementation. Furthermore, apigenin administration significantly stimulated loricrin, aquaporin‐3, filaggrin, hyaluronic acid, hyaluronic acid synthase, and human β‐defensin expression in HaCaT cells (Park et al. 2020). Moreover, apigenin alleviated lipopolysaccharide‐induced IL‐6, IL‐1β, TNF‐α, and NF‐κB expression while improving HO‐1, Nrf2, and GSK3β signaling pathways to inactivate lipopolysaccharide‐induced microglia (Chen et al. 2020). Similarly, apigenin and curcumin, in combination and nanoparticle formulations, revealed anti‐inflammatory potential by reducing iNOS, IL‐6, and TNF‐α expression in lipopolysaccharide‐stimulated RAW 264.7 macrophage cells (Singh et al. 2023; Wang et al. 2022; Hong et al. 2021). Their anti‐inflammatory potential is demonstrated in Figure 3.

Previously, dimethylglyoxal apigenin (an apigenin‐Maillard reaction product) demonstrated a similar anti‐inflammatory effect in RAW 264.7 cells (Zhou et al. 2019). Acrolein, an environmental pollutant, enhances inflammation and inflammatory markers in human umbilical vein endothelial cells. The effect of apigenin and apigenin‐7, 4‐O‐octanoate was evaluated by Yu et al. (2022), and it was depicted that apigenin and apigenin‐7, 4‐octanoate alleviated IL‐1β, IL‐6, TNF‐α, NLRP3, high‐mobility group box 1 (HMGB1), NF‐κB, and TLR4 levels to attenuate acrolein‐induced inflammation.

## 7 Antimicrobial Activity

Beneficial and harmful microbes reside in large intestines and are essential for regulating normal metabolic processes. Any disturbance in their balance disturbs the homeostatic mechanism, ultimately leading to the pathogenesis of various complications. Plants, particularly *S. coccinea* , demonstrated antimicrobial attributes against multiple bacterial and fungal strains. The antibacterial activity of the silver nanoparticles of *S. coccinea* was investigated by the disc diffusion method against human pathogenic bacteria, such as *Bacillus species* (MTCC 511), *Proteus vulgaris* (MTCC 425), *Bacillus subtilis* (MTCC 121), and *Staphylococcus aureus* (MTCC 96). It has been depicted that the aqueous extract of *S. coccinea* silver nanoparticles (50 μL) has a higher zone of inhibition against the respective strains, *Bacillus species* (19 nm), *Proteus vulgaris* (21 nm), *Bacillus subtilis* (20 nm), and *Staphylococcus aureus* (9 nm), as compared to other concentrations, i.e., 20, 30, and 40 μL (Rajendran and Prabha 2020). Similarly, the oil of *S. coccinea* revealed antibacterial activity against *E. coli* , *S. aureus* , *Klebsiella pneumoniae*, and *B. subtilis* (Mohan et al. 2019). The antifungal activity of the methanol extract of dried aerial parts of *S. coccinea* against *Candida species* and *Mycobacterium tuberculosis* species was assessed by the microdilution method. The results showed that *S. coccinea* has comparatively lower antifungal activity (31.25 and 125 μg/mL) against different strains of *Candida*; however, it showed maximum inhibition (MIC = 125 μg/mL) against *Mycobacterium tuberculosis* (Koncz et al. 2021). Apigenin also exhibits antimicrobial potential, which is evidenced by various studies in Table 3.

**TABLE 3:** Antimicrobial potential of apigenin against various bacterial and fungal strains.

Species	Model	Strain	Mechanism of action	References
Oral bacteria	Broth dilution method	*S. mutans* (ATCC 25175), *S. sanguinis* (ATCC 10556), *S. sobrinus* (ATCC 27607), *S. ratti* (KCTC 3294), *S. criceti* (KCTC 3292), *S. anginosus* (ATCC 31412), *S. gordonii* (ATCC 10558), *A. actinomycetemcomitans* (ATCC 43717), *F. nucleatum* (ATCC 51190), *P. intermedia* (ATCC 49049), and *P. gingivalis* (ATCC 33277)	MIC_50_ of 25 and 12.5 μg/mL for the respective *S. mutans* (ATCC 25175), *S. sanguinis* (ATCC 10556), *S. sobrinus* (ATCC 27607), *S. ratti* (KCTC 3294), *S. criceti* (KCTC 3292), *S. anginosus* (ATCC 31412), *S. gordonii* (ATCC 10558), *A. actinomycetemcomitans* (ATCC 43717), *F. nucleatum* (ATCC 51190), *P. intermedia* (ATCC 49049), and *P. gingivalis* (ATCC 33277)	Cha et al. (2016)
*Escherichia coli*	Microdilution method	*MG1655*	MIC (2.5 μg/mL), ↑reactive oxygen species (ROS), ↑superoxide anions (O_2_^−^)	Kim et al. (2020)
	Disc diffusion method	ATCC 25922	Zone of Inhibition = 10 mm	Liu et al. (2013)
	Broth microdilution method	ATCC 14948	MIC = > 400 μg/mL	Lucarini et al. (2015)
	Agar dilution method	JM109	MIC = 200 μg/mL	Wu et al. (2008)
	Microdilution method	ATCC 35218	MIC = 4 μg/mL	Ozçelik et al. (2011)
Methicillin‐resistant *Staphylococcus aureus*	Microdilution method and time‐kill assay	Methicillin‐resistant *Staphylococcus aureus*	FIC indices of 0.18–0.47, ↓colony count to 99%	Akilandeswari and Ruckmani (2016)
*Acinetobacter baumannii*	Broth microdilution method	RSKK 02026	MIC = 2 μg/mL	Ozçelik et al. (2011)
*Enterobacter aerogenes*	Broth dilution method	ATCC 13048	MIC = 64 μg/mL	Basile et al. (2000)
	Disc Diffusion method	MTCC111	Inhibition zone = 14 mm	Nayaka et al. (2014)
*Enterobacter cloacae*	Broth dilution method	ATCC 10699	MIC = 64 μg/mL	Basile et al. (2000)
*Enterococcus faecalis*	Broth microdilution method	ATCC 19433	MIC = 400 μg/mL	Lucarini et al. (2015)
*Helicobacter pylori*	Agar Dilution method	ATCC 43504	MIC = 25 μg/mL	Wu et al. (2008)
*Klebsiella pneumoniae*	Broth dilution method	ATCC 10031	MIC = 128 μg/mL	Basile et al. (2000)
	Disc diffusion method	MTCC109	Zone of inhibition = 10 mm	Nayaka et al. (2014)
	Microdilution method	RSKK 574	MIC = 8 μg/mL	Ozçelik et al. (2011)
*Pseudomonas aeruginosa*	Broth dilution method	ATCC 27853	MIC = 400 μg/mL	Lucarini et al. (2015)
	Disc diffusion method		Inhibition zone = 10 mm	Liu et al. (2013)
		MTCC424	Zone of inhibition = 12 mm	Nayaka et al. (2014)
	Microdilution method	ATCC 10145	MIC = 2 μg/mL	Ozçelik et al. (2011)
*Staphylococcus aureus*	Broth Microdilution method	ATCC 10832	MIC = 1024 μg/mL	Dong et al. (2013)
		strain Mu50	MIC = 4 μg/mL	Morimoto et al. (2015)
	Disk diffusion method	ATCC 25923	Inhibition zone = 11 mm	Liu et al. (2013)
	Microdilution method		MIC = 16 μg/mL	Ozçelik et al. (2011)
*Streptococcus pyogenes*	Broth microdilution	ATCC 12344	MIC = 4 μg/mL	Mamadalieva et al. (2011)
*P. mirabilis*	Disc Diffusion method	MTCC425	Inhibition zone = 19 mm	Nayaka et al. (2014)
	Microdilution method	ATCC 7002	MIC = 4 μg/mL	Ozçelik et al. (2011)
*S. typhimurium*	Disc Diffusion method	MTCC98	Inhibition zone = 17 mm	Nayaka et al. (2014)
*Candida albicans*	Microdilution method	ATCC 10231	MIC = 8 μg/mL	Ozçelik et al. (2011)
	Two‐fold serial dilution method	ATCC90028	MIC = 5 μg/mL	Lee et al. (2018)
*Candida parapsilosis, Malassezia furfur*, and *Trichophyton rubrum*	Two‐fold serial dilution method	ATCC22019, KCTC7744, and KCTC 6345	MIC = 5 μg/mL	Lee et al. (2018)

## 8 Cardioprotective Effect

Cardiovascular disorders are the leading cause of mortality worldwide, accounting for ~31% of overall deaths (Wells et al. 2020). Ischemic heart disease (IHD) is an enormous global health concern, contributing to ~40% of demises worldwide. Cardiac damage, arrhythmias, and cardiac arrest are attributed to ischemia–reperfusion injury (Furman et al. 2019). Traditional Chinese Medicine (TCM) uses salvia species, especially *S. coccinea* , extensively as Danshen to treat heart issues (Tu et al. 2022). *S. coccinea* freeze‐dried extract provided a cardio‐protective effect in a mouse model of ischemia–reperfusion injury. The extract showed a dose‐dependent response, and ~50 mg extract improved post‐ischemic contractile function and adverse chronotropic effect. Additionally, the similar dose protected cardiac functions by improving LVDP recovery (early: 51.4% ± 9.7%, later: 38.6% ± 8.9%). Furthermore, the heart rate was notably reduced (90.0 ± 7.0 beats/min), and the dose–response curve was reversed, exhibiting a U‐shaped curve. At 50 mg, adenosine partially decreased LVDP recovery (9.5% ± 3.2% with naloxone vs. 15.5% ± 5.8% with adenosine), while naloxone entirely removed it (Nyaga et al. 2018). An imbalance in the free radicals and antioxidants produced oxidative stress and, ultimately, myocardial infarction (MI). A study was conducted to examine the cardio‐protective effects of *S. coccinea* leaf aqueous extract (AESL) in Albino Wistar rats using isoproterenol‐induced MI. AESL (600 mg/kg body weight) lowered serum cardiac enzyme levels (creatine kinase, cardiac Troponin T, cardiac Troponin I, Lactate dehydrogenase, aspartate aminotransferase, and Alanine aminotransferase) in addition to improving ECG, thereby protecting heart tissues from histopathological modifications as well as aiding in attenuating MI (Sundaramoorthy and Shanmugam 2023).

The cardioprotective property of *S. coccinea* is due to its potent bioactive compounds, particularly apigenin, that modulate oxidative and inflammatory markers. Multiple studies have proved the protective effect of apigenin against cardiovascular disorders. For instance, it has been revealed that apigenin attenuates the incidence of hypertension by reducing reactive oxygen species (ROS), oxidative stress, lipid peroxides, and inflammatory cytokines, such as IL‐6, IL‐10, IL‐1β, MCP‐1, and TNF‐α. Moreover, the AMPK/SIRT1 pathway was regulated alongside NO production and ACE inhibition by apigenin supplementation (Gao et al. 2021; Lin et al. 2020). Moreover, apigenin contributes to alleviating lipid accumulation, body weight, total cholesterol (TC), triglycerides (TG), and low‐density lipoprotein (LDL) levels, consequently managing dyslipidemia and cardiovascular disorders (Wu et al. 2021; Clayton et al. 2021). Atherosclerosis is a risk factor that aggravates the progression of cardiovascular disorders. Apigenin has the potential to manage atherosclerosis by repressing proinflammatory cytokines, plaque formation, and the TLR‐4/NF‐κB signaling pathway (Wang et al. 2020; Ren et al. 2018). Recently, gold nanoparticles were prepared using apigenin, and their effect against doxorubicin‐induced cardiotoxicity was studied among male rats (*n* = 40). It has been revealed that gold nanoparticles significantly suppressed myocardial apoptosis by regulating Bax, Bcl‐2, and caspase‐3 expression (Sharifiaghdam et al. 2023). Li et al. (2023) observed a similar cardioprotective effect of apigenin, which depicted that apigenin enhanced the expression of Sirt1 and Atf5 to promote cardiac health. Furthermore, the effect of apigenin and apigenin‐enriched zinc oxide nanoparticles was investigated against cisplatin‐induced cardiotoxicity among male Wistar rats (*n* = 32). The results revealed that apigenin nanoparticles have more cardioprotective potential than apigenin alone. Apigenin nanoparticles improve lipid profile, antioxidant activities, and inflammatory markers, promoting cardiac functions (Alaqeel 2025).

**FIGURE 4:**
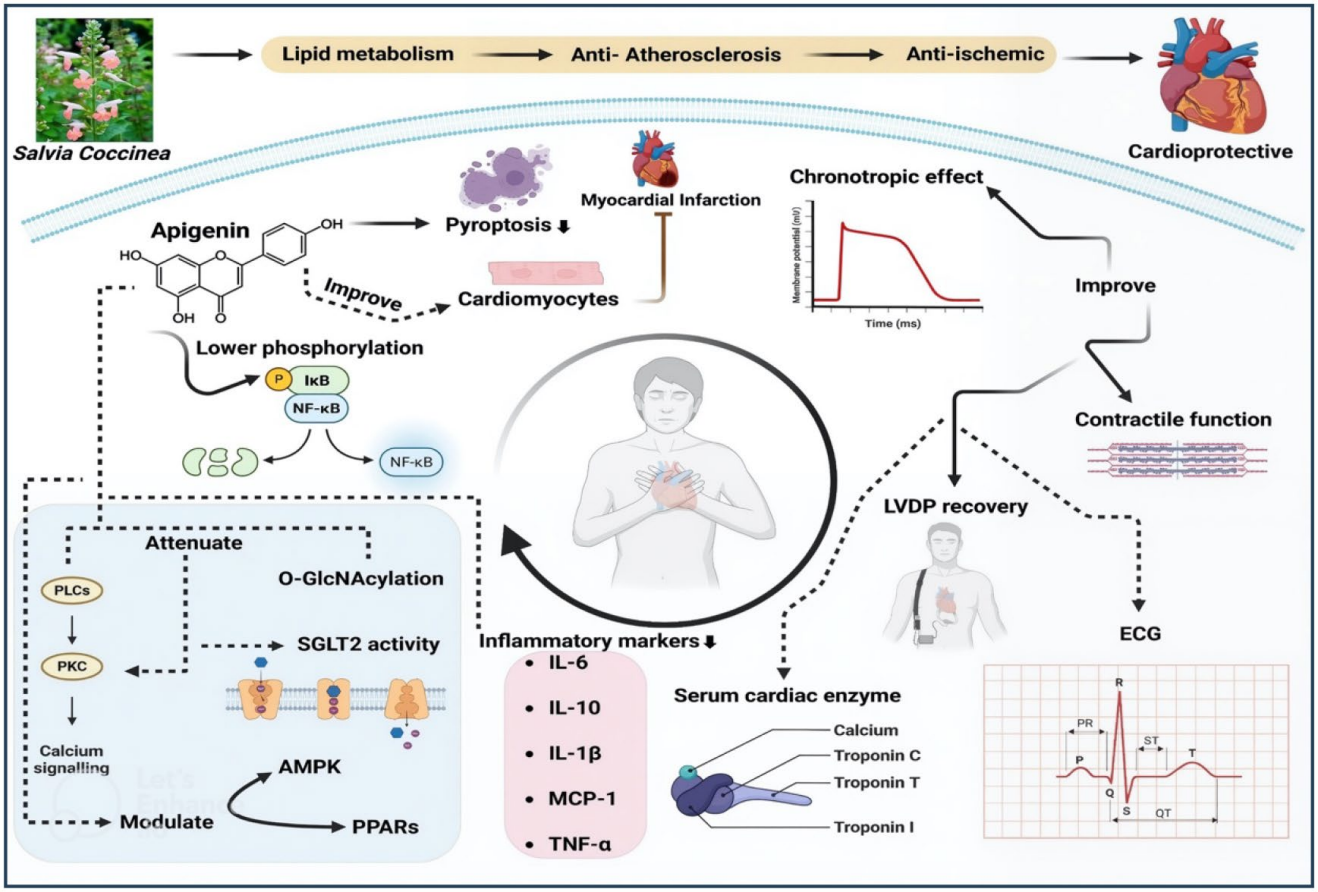
Cardioprotective potential of *S. coccinea* and apigenin.

A study was conducted to evaluate the impact of apigenin against doxorubicin‐induced cardiotoxicity, which showed that apigenin supplementation lowered pyroptosis levels and the phosphorylation of NF‐κB p65. Whereas apigenin improved the phosphorylation of GSK‐3β, thereby attenuating cardiotoxicity (Wang, Yan, et al. 2024; Wang, Yu, et al. 2024). Furthermore, the mouse models evaluated the cardioprotective mechanism of apigenin against oxidative stress, isoproterenol, and hypoxia/reoxygenation‐induced myocardial injury. The results showed that apigenin improved cardiomyocyte shape and physiology by down‐regulating apoptotic pathways and inflammation and up‐regulating the SIRT1 signaling pathway (Xu et al. 2023). Another study revealed that apigenin and astilbin synergistically protected cardiac functions by attenuating oxidative stress, inflammatory pathways (NF‐κB, JNK, and MAPK), O‐GlcNAcylation, PKC, and SGLT2 activity. Additionally, apigenin modulated the AMPK and PPAR pathways, further reducing cellular damage (Dhiman et al. 2024). Furthermore, apigenin alleviated myocardial infarction‐induced cardiomyopathy injury and ischemia/hypoxia‐induced myocardial injury by regulating parkin‐mediated mitochondrial autophagy (Wang et al. 2020) and by promoting apoptotic pathways (Li et al. 2020), respectively. Aluminum phosphide causes toxicity in cardiomyocytes, and to reduce its progression and toxicity, apigenin (5–100 μM) was explored, which confirmed the protective effect of apigenin against aluminum phosphide‐induced toxicity. This effect was due to its capability of alleviating cytotoxicity and mitochondrial fluctuations (Jahedsani et al. 2020). Figure 4 illustrates the cardioprotective potential of *S. coccinea* and apigenin.

## 9 Neuroprotection

Individual health regulates the body's physiological functions and comprises physical, cognitive, social, and emotional well‐being. Various factors, such as genetics, environmental factors, lifestyle choices, and eating and sleep patterns, influence normal health. Cognitive health is equally important for individuals as physical, social, and emotional health. Medicinal plants and their phytochemicals, e.g., apigenin, have improved overall health as some studies demonstrated the neuroprotective effect of apigenin supplementation (20 and 40 mg) against mild traumatic brain injury. Apigenin reduced the levels of luminol and lucigenin and enhanced IL‐10 (an anti‐inflammatory cytokine) levels (Kuru Bektaşoğlu et al. 2023). Moreover, the neuroprotective mechanism of apigenin was investigated in preclinical studies which demonstrated that apigenin reduced pro‐inflammatory cytokines and microglia activation by repressing ROCK‐1, iNOS, COX‐2, toll‐like receptor‐4, NF‐κB, NLRP3 inflammasome, and CD‐11b‐positive cells as well as modulating miR‐15a, brain‐derived neurotrophic factor (BDNF), p‐ERK1/2, and p‐CREB (Olasehinde and Olaokun 2024). Further studies evidencing the neuroprotective potential of apigenin are presented in Table 4.

**TABLE 4:** Experimental trials regarding the neuroprotective potential of apigenin.

Studies	Neurological disorder	Route and dosage	Mechanism of action	References
In vivo	Alzheimer's disease	Orally and 1 μM apigenin	↑Neuronal integrity, ↓Microglial activation, ↓ CD68 expression, ↓IL‐6, gp130, and OX42 expression, and ↑ Brain‐derived neurotrophic factor (BDNF)	Dourado et al. (2020)
In vivo	Alzhemier's Disease	Orally and 50 mg/kg body weight of apigenin	↓Hyperphosphorylation of tau levels, ↓ expression of GSK‐3α, ↓ mRNA level of β secretase (BACE1)	Alsadat et al. (2021)
In vitro and in vivo	Cerebral ischemia/reperfusion injury	10 μg/mL	↑Cell viability, ↑MMP, ↓ROS, ↓apoptosis	Ling et al. (2020)
In vivo	Alzheimer's disease		↑Tropomyosin‐related kinase B Tyr516 and Tyr817, ↓ cAMP‐response‐element binding protein (CREB), ↑ Bcl‐2 and BDNF	Chiu et al. (2023)
In vivo	Streptozocin‐induced depression	Oral and 20 mg/kg body weight apigenin	↑Energy metabolism, ↑immune system, ↓NLRP2 and TLR4 expression, ↑AMPK expression, ↑coenzyme Q10	Bijani et al. (2022)
In vivo	Hypoxic–ischemic brain injury		↓Infarct volume, ↓cerebral edema, ↓inflammation, ↓apoptosis, PI3K/Akt/Nrf2 pathway	Fu et al. (2021)
Pre‐clinical	Neuroinflammation		↓Pro‐inflammatory cytokines, ↓CD‐11b‐positive cells, ↓ROCK‐1, NLRP3, iNOS, TLR4, NF‐κB, and COX‐2 expression, ↑BDNF, p‐ERK1/2, and miR‐15a	Olasehinde and Olaokun (2024)
In vitro	Multiple sclerosis	20 μM	↓T‐bet, Interferon (IFN)‐γ, IL‐17 expression, ↑TGF‐β, IL‐10, and FoxP3	Ginwala et al. (2021)
In vivo	Parkinson's disease	Oral and 25 and 50 mg/kg	↓SOD, MDA, nitrite, TNF‐α, IL‐6, IL‐1β, and caspase‐1 levels, ↑CAT (catalase) and GSH (glutathione), ↑NF‐κB and Nrf2 expression	Patel and Singh (2022)
In vivo	Parkinson's disease	50 mg/kg body weight	↓TNF‐α, ↓IL‐1β, ↓IL‐6, ↓TGF‐β, and ↑IL‐10	Yarim et al. (2022)
In vivo	Alzhemier's disease	110 mg/kg body weight	↓Iba‐1, ↓hippocampal microglia	Chesworth et al. (2021)
In vivo	Oxidative stress‐induced behavioral modifications	150 mg/kg body weight	↓Oxidative damage, ↑sedative effect	Almzaien et al. (2022)
In vivo	Monosodium glutamate‐induced neuronal damage	Orally, 20 mg/kg body weight	↑Acetylcholinesterase activity, ↑dopamine, serotonin, and norepinephrine, ↓monoamine oxidase, ↓MDA levels, ↑GSH, SOD, and CAT levels, ↓pro‐inflammatory cytokines (NO, IL‐1b, and TNF‐α)	Albrakati (2023)
In vivo	Alzhemier's disease		↓NF‐κB, ↑SOD, ↑GPx (glutathione peroxidase), ↓MDA, ↑BDNF and pCREB, ↓caspase‐3	Zhang et al. (2025)
In vivo	Methylmercury‐induced neurotoxicity	40 and 80 mg/kg body weight	↑Learning, cognition, and motor skills, ↓c‐JNK and p38MAPK signaling pathway, ↓Bax, Bcl‐2, caspase‐3, TNF‐α, and IL‐1β	Yadav et al. (2022)
In vivo	Lipopolysaccharide‐induced neurotoxicity and cognitive impairment	40 mg/kg body weight	↑Mitochondrial sirtuin‐3 (SIRT3) activity, ↑ eroxisome proliferator‐activated receptor γ (PPARγ), ↑optic atrophy‐1, ↑mitofusin 2, ↑Parkin expression, ↑microtubule‐associated protein 1 light chain 3 II/I ratio (LC3II/I)	Ahmedy et al. (2022)
In Vitro	Oxidative Stress‐induced Neuronal Apoptosis		↓Cytochrome C, ↓Bax, ↓Bcl‐2, ↓cleaved caspase‐3, ↓oxidative stress	Kim, Cho, et al. (2021); Kim, Kim, et al. (2021)
In vivo and in vitro	Retinal ischemia/reperfusion‐induced retinal ganglion cells' degeneration		↑Retinal inner plexiform layer, ↓apoptosis, ↓lactate dehydrogenase (LDH) release, ↑Bax and Bcl‐xL expression, ↑ mitochondrial membrane potential (MMP), ↑OPA1, MFN2, and DRP1 expression	Wu et al. (2024)
In vivo	Scopolamine‐induced cognitive dysfunction	100 and 200 mg/kg body weight	↓Lipid peroxidation, ↓Bax/Bcl‐2 ratio, ↓caspase‐3, presenilin 1 and 2 levels, ↑BDNF and tropomyosin receptor kinase B (TrkB) expression	Kim, Cho, et al. (2021); Kim, Kim, et al. (2021)
In vivo	Lead acetate‐induced toxicity in cerebellum	20 mg/kg body weight	↓Oxidative stress, ↓IL‐6, ↓TNF‐α, ↑IL‐4, ↑IL‐10	Alfwuaires et al. (2023)
In vivo	Methotrexate (MTX)‐induced neurotoxicity	20 mg/kg body weight	↓IL‐1β, ↓MDA, ↓caspase‐3, ↑GSH, ↑miR‐15a expression, ↓ROCK‐1, ↑ERK1/2 and cAMP response element‐binding protein (CREB) phosphorylation, ↑BDNF	Taha et al. (2023)
In vivo	Corticosterone‐induced depression		↑Dopamine, ↑norepinephrine, ↑CREB, ↑BDNF, ↓apoptosis	Zhang et al. (2023)
In vivo	Hyperglycemia‐associated amnesia		↑Spatial learning and cognition, ↓blood glucose levels, ↓oxidative stress, ↓ acetylcholinesterase activity, ↑Nrf2/ARE expression	Haridevamuthu et al. (2024)
In vitro	Cerebral stroke	1 μM	↓ROS production, ↓apoptotic pathway, ↓p53 expression	Miraee et al. (2021)
In vivo	Proton pump inhibitor‐induced cognitive impairment	25, 50, and 100 mg/kg body weight	↓GSK‐3β pathway, ↑cognition, ↓oxidative stress, ↓inflammatory markers (TNF‐α, IL‐10, and IL‐1β)	Bhratee et al. (2025)
In vivo	Prenatal valproic acid‐induced autism spectrum disorder	50 mg/kg body weight	↓MDA, ↓IL‐6, ↓TNF‐α, ↑GPx, ↑SOD	Farbin et al. (2024)
In vivo	Alzhemier's disease	25, 50, 75, and 100 μM	↓Oxidative stress, ↓ acetylcholinesterase activity, ↓ Aβ‐42 accumulation	Siddique et al. (2022)
*In silico*	Ischemic stroke		↑Blood–brain barrier integrity, ↑JAK/STAT and TLR signaling pathways	Wang, Yan, et al. (2024), Wang, Yu, et al. (2024)
In vivo	Pentylenetetrazole (PTZ) kindling associated cognitive impairment	10 and 20 mg/kg body weight	↓Anxiety, ↑BDNT, ↑CREB	Sharma et al. (2020)
In vivo	Seizures	100 and 150 mg/kg body weight	↑GABA_A_R‐immunoreactivity, ↑parvalbumin‐immunoreactivity	Socała et al. (2025)
In vivo	Amyotrophic lateral sclerosis		↓Weight loss, ↑motor functions, ↑ALDH1A2	Liang et al. (2024)
In vitro	Tetrabromobisphenol A‐induced cytotoxicity		↑Cell viability, ↓apoptosis, ↓IL‐1β and nitrite, ↓oxidative stress, ↓NOX4, ↑Nrf2, ↓Akt and ERK	Choi et al. (2023)
In vivo	Ischemic stroke	30, 60, and 120 mg/kg body weight	↓Cerebral edema, ↓parthanatos, ↓PARP1/AIF pathway, ↓oxidative stress, ↓MDA, PARP1, 53BP1, and cleaved caspase‐3, ↑RAD51 and BRCA1	Ping et al. (2024)

## 10 Anti‐Diabetic Effect

Diabetes mellitus is a global public health concern characterized by impaired metabolism and increased blood glucose concentrations. Medicinal plants, particularly *S. coccinea* , have gained popularity due to their antidiabetic potential. *S. coccinea* and its phytochemicals, such as terpenoids, polyphenols, and flavonoids, contribute to the hypoglycemic effect (Sudaramoorthy et al. 2021). Further studies provided evidence that supports the antidiabetic potential of *S. coccinea* . For instance, Rodriguez (2021) conducted a study to explore the hypoglycemic effect of *S. coccinea* in diabetic rats. The results showed a significant reduction in blood sugar levels during fasting and improved insulin sensitivity. Another study revealed that *S. coccinea* suppressed alpha‐amylase activity, demonstrating its utilization in regulating postprandial glucose levels. Moreover, the aqueous extract of *S. coccinea* (AESL) has attenuated hyperglycemic‐induced ROS production and NF‐κB dependent pro‐inflammatory gene expression in streptozotocin‐induced diabetic rats (Sudaramoorthy et al. 2021). Moreover, the antidiabetic effect of its bioactive compound, apigenin, was extensively studied and described here in detail.

Apigenin can lower blood glucose levels by inhibiting α‐amylase activity, cholesterol levels, and apoB/apoA1 ratio. Furthermore, it improved glucose tolerance and GLUT‐4 levels, thereby regulating glucose uptake by the muscles and adipose tissue cells (Barky et al. 2020). Additionally, the hypoglycemic effect of apigenin was investigated against dexamethasone and high‐glucose‐induced insulin resistance (IR) HepG2 cells. The results showed that apigenin elevated glucose utilization and glycogen synthesis by modulating GLUT4 and GSK‐3β and discouraging ROS and advanced glycation end products (AGEs) production (Miao, Zhang, et al. 2023; Miao, Cheong, et al. 2023). Prolonged diabetes mellitus stimulates oxidative stress, inflammation, cell apoptosis, and ultimately endothelial dysfunction. A study was conducted to alleviate the progression of endothelial dysfunction by apigenin supplementation in C57BL/6J mice and cultured rat aortic endothelial cells. It has been found that oral administration of apigenin (25 mg/kg/day) enhanced endothelium relaxation by upregulating adenosine 5′‐monophosphate‐activated protein kinase (AMPK), phosphatidyl inositol 3‐kinase (PI3K), nuclear factor erythroid 2‐related factor 2 (Nrf2), protein kinase B (Akt), heme oxygenase 1 (HO‐1), and endothelial nitric oxide synthase (eNOS) expression (Miao, Zhang, et al. 2023; Miao, Cheong, et al. 2023). Recently, Li et al. (2024) demonstrated that apigenin regulates early wound healing in diabetic mice by elevating macrophage miR‐21 expression. Moreover, apigenin attenuated α‐glucosidase activity to reduce fasting blood sugar levels and insulin resistance (Liu et al. 2024; Shahab et al. 2024).

Type 1 diabetes mellitus, an autoimmune disorder, leads to podocyte loss, excessive endothelial filtration, and gonadal dysfunction that disturbs normal reproductive health. Apigenin (0.78 mg/kg/day) was supplemented in male Wistar rats (*n* = 28) of 180–220 g for 10 days to evaluate its alleviating potential against diabetes‐induced genitourinary complications. It was revealed that apigenin enhanced body weight, renal and reproductive function, and attenuated glucose levels by decreasing dynamin‐related protein 1 (Drp1) expression (Khaled et al. 2024; Sherif et al. 2024). The underlying antidiabetic and pancreatic β‐cell insulin secretory potential of apigenin was studied in INS‐ID β‐cell lines. The results revealed that apigenin suppressed endoplasmic reticulum stress signaling protein, cleaved caspase‐3 expression, and thapsigargin‐induced thioredoxin‐interacting protein (TXNIP) expression, thereby facilitating insulin secretion by the pancreatic β‐cells (Ihim et al. 2023). Apigenin revealed antidiabetic potential by down‐regulating protein tyrosine phosphatase 1β expression, cellular DNA damage, β‐cell apoptosis, and protein carbonylation (Javadi and Sobhani 2024). Fetuin‐A induces insulin resistance by binding insulin receptors; therefore, the protective mechanism of apigenin was explored on insulin resistance in Huh7 cells. The results depicted that Fetuin‐A expression was slowed by apigenin, which was attributed to lowered ROS‐mediated casein kinase 2α and NF‐κB expression. Furthermore, apigenin improved GLUT2 and Akt expression, consequently improving obesity‐induced insulin resistance (Hsu et al. 2024). Moreover, Ogura et al. (2020) demonstrated that apigenin attenuated the pathogenesis of diabetic kidney disease by suppressing CD38 expression and elevating Sirt3 activity.

Apigenin potential was evaluated against high‐fat diet‐induced nephropathy in rats by supplementing 50 mg/kg body weight of apigenin for 3 months. It has been exposed that apigenin lowered the progression of high‐fat induced nephropathy by enhancing Nrf2, GSH, SOD, and CAT activity, as well as by attenuating the activity of MDA, TNF‐α, IL‐6, NF‐κB, TGF‐β1, Bax, and caspase‐3 (Aldayel 2022). Furthermore, the apigenin anti‐diabetic potential was investigated among streptozotocin‐induced diabetic rats by administering ~50 mg/kg body weight apigenin. The results revealed that apigenin supplementation significantly reduced blood glucose levels and improved body weight and lipid profile (Anandan and Urooj 2021). The renal protective potential of apigenin was explained by Wu et al. (2021), who showed that apigenin alleviated insulin resistance and lipid accumulation in palmitate‐induced HepG2 cells and high‐fat diet‐fed mice by downregulating SREBP‐1c, SREBP‐2, and ERS expression. Furthermore, apigenin attenuated the pathogenesis of hyperuricemia and renal injury by lowering GLUT‐9, urate anion transporter 1 (URAT1), IL‐6, phospho‐janus kinase 2 (P‐JAK2), JAK2/STAT3, and Wnt/β‐catenin pathway (Liu et al. 2022; Li, Yang, et al. 2022; Li, Wu, et al. 2022).

## 11 Wound Healing

Skin is the largest organ of the human body, constituting three principal layers, namely the epidermis, dermis, and hypodermis, which maintain skin integrity and aid in regulating its standard functionality. It mainly acts as a barrier that inhibits environmental contaminants and other microbial pathogens from entering the body and protects against serious health concerns (Uberoi et al. 2024; Mahmoud et al. 2024). Wound healing from injury, surgery, or any other condition occurs in four steps: homeostasis, inflammation, proliferation, and remodeling. Platelets reach the bleeding site and stop bleeding within minutes to regulate homeostasis. Moreover, injury results in the release of transforming growth factors (TGFs), platelet‐derived growth factors (PDGFs), and damage‐associated molecular patterns (DAMPs). Later, innate immune cells are activated during the inflammation phase, and keratinocytes move toward the wound to re‐epithelize the wound.

Furthermore, keratinocytes divide to improve epidermal thickness and regenerate the dermis. New blood vessels are formed during the proliferation phase to ensure blood and nutrient supply. The mature, organized, cross‐linked collagen fibers and deposited extracellular matrix (ECM) protein strengthen epithelium and structural integrity (Choudhary et al. 2024). Medicinal plants, particularly *S. coccinea* , modulate wound healing by repairing damaged cells and helping in cell inflammation, proliferation, and regeneration. The in vivo wound healing activity of *S. coccinea* was investigated among healthy rats (*n* = 105) administered with wound ablation. The results showed that *S. coccinea* enhanced the regeneration of blood vessels and fibroblasts, demonstrating its wound‐healing properties. Furthermore, Güzel et al. (2019) assessed the in vivo wound healing potential of *S. coccinea* . The ethanolic fraction (0.5% and 1%) of *S. coccinea* was subjected to streptozotocin‐induced diabetic rats, which resulted in re‐epithelialization, angiogenesis, and decreased dermal inflammation. Furthermore, the hydroalcoholic extract of *S. coccinea* has significantly high wound‐healing properties due to rosmarinic acid content. Later, *S. coccinea* extract increased wound closure (60% re‐epithelialized) on the fourth day in a positive control group (Scrima et al. 2020).

## 12 Larvicidal Properties

Rodents and other insects are harmful to the productivity of crops besides deteriorating their quality. Although insecticides and pesticides are commercially available to reduce insect and pest attacks, their adverse consequences on the nutritional, phytochemical, and therapeutic profile urge scientists and agricultural workers to use alternative substances. *S. coccinea* , a medicinal plant, can kill and reduce the production of harmful insects in addition to its curative properties. Mathew and Thoppil (2011) depicted the larvicidal properties of mosquitoes extracted from the essential oils of Salvia species, including *S. elegans* , *S. coccinea* , and *S. splendens* . Spathulenol (38.73%) and caryophyllene (10.32%) from *S. elegans* ; β‐cubebene (22.9%) and caryophyllene (12.99%) from *S. splendens* Blue Ribbon; phytol (41.46%) and cyclooctasulfur (24.88%) from *S. splendens* Scarlet Sage Red are the main components derived from the essential oils. Another study carried out by Alejandro et al. (2020) explored the insecticidal potential of the chloroform extract of Salvia species, and their results revealed the insecticidal activity at an LC50 of 200 ppm and insectistatic effects at 80 ppm. Moreover, Ali et al. (2015) demonstrated the chemical compositions of different essential oils in Salvia species. The mosquito‐repelling properties of *Salvia leucantha* and *Salvia elegans* oils were equally effective as DEET in biting‐deterrent bioassays. Larvicidal activity is present in all species of Salvia, especially *S. coccinea* , except *S. apiana* and *S. leucantha* .

## 13 Industrial Applications

*S. coccinea* , one of the sustainable sources of bioactive compounds, has various therapeutic health benefits that reduce the disease burden. *S. coccinea* is an enriched source of antioxidants and can be applied in food products, pharmaceutical industries, and cosmetic industries to facilitate individuals (Grzeszczuk et al. 2018). The medicinal attributes followed by several bioactive compounds make it a potential commodity to overwhelm personal food choice behaviors and attitudes. These properties satisfy the nutritional needs of individuals; therefore, compromising nutritional adequacy.

## 14 Limitations, Future Perspectives, and Conclusions

Medicinal plants are the cornerstone of both modern and conventional healthcare sectors, as they are incorporated into the pharmaceutical and nutraceutical industries to prepare synthetic drugs and nutraceuticals. *S. coccinea* is widely distributed worldwide and is renowned for its versatile morphology. The unique nutritional profile enables food industries to utilize it as a functional ingredient in the manufacturing of products that meet the nutritional needs of individuals. Moreover, its phytochemical ingredients, such as apigenin, luteolin, camphor, cineol, and phytol, enhance the curative significance. Moreover, it attenuates oxidative stress by neutralizing free radicals, which aids in ameliorating the pathogenesis and prevalence of cancers, including breast, ovarian, colorectal, gastric, and pancreatic cancer. Additionally, the antidiabetic, antimicrobial, anti‐inflammatory, and wound‐healing properties of *S. coccinea* have also been validated. Moreover, its cardio‐ and neuro‐protective properties are attributed to its potential to lower inflammatory markers (IL‐6, IL‐8, IL‐1β, IFN‐γ, NF‐κB, COX‐2, and CRP), as well as to improve lipid profile. Furthermore, the modulation of Bax, Bcl‐2, caspase‐3, Sirt1, and GSK‐3β expression alleviates the incidence of myocardial infarction, ischemic stroke, and coronary heart disease, thereby improving cardiac health. There are limited studies available regarding the health attributes of *S. coccinea* ; therefore, scientists are exploring comprehensive and long‐term rodent, pre‐clinical, clinical, and experimental studies to validate their therapeutic profile. Additionally, experiments are recommended to explore its active compounds and their mechanism of action at the molecular level. Although apigenin is effective in managing multiple disorders, its bioavailability is a significant concern for both individuals and healthcare providers, as reported in Table 5. Factors such as food matrix, digestibility, bio‐accessibility, transporter, molecular structure, and delivery route influence the bioavailability of apigenin. Its bioavailability could be improved by adopting different strategies, such as phospholipid complexes, nanoparticles, polymeric carriers, liposomes, self‐nanoemulsifying systems, glycosylation, enzyme or transporter modulation, thereby improving systemic circulation and ultimately enhancing therapeutic significance. Furthermore, *S. coccinea* could be employed as an important ingredient in the food industry to prepare therapeutic food products, not only for reducing disease severity but also to improve the nutritional status of individuals.

**TABLE 5:** List of abbreviations.

DPPH	2,2‐diphenyl‐1‐picrylhydrazyl
IC_50_	Inhibitory concentration 50
NF‐κB	Nuclear Factor kappa‐light‐chain‐enhancer of activated B cells
EMT	Epithelial–mesenchymal transition
PARP	Poly (ADP‐ribose) polymerase
ERK	Extracellular signal‐regulated kinase
CDK‐1	Cyclin‐dependent kinase‐1
Bax	Bcl‐2‐associated X protein
IFN‐γ	Interferon‐gamma
PD‐L1	Programmed death ligand 1
Bcl‐2	B‐cell lymphoma 2
IL‐6	Interleukin‐6
CRP	C‐reactive protein
MMP	Matrix metalloproteinases
MAPK	Mitogen‐activated protein kinase
TLR4	Toll‐like receptor 4
COX‐2	Cyclo‐oxygenase‐2
LPO	Lipid peroxidation
TGF‐β1	Transforming growth factor‐beta 1
SOD	Superoxide dismutase
CAT	Catalase
MCP‐1	Monocyte chemoattractant protein 1
iNOS	Inducible nitric oxide synthase
TNF‐α	Tumor necrosis factor‐alpha
Nrf2	Nuclear factor erythroid 2‐related factor 2
GSK3β	Glycogen synthase kinase 3‐beta
NLRP3	NOD‐like receptor family, pyrin domain containing 3
HMGB1	High‐mobility group box 1
IHD	Ischemic heart disease
TCM	Traditional Chinese medicine
MI	Myocardial infarction
ECG	Electrocardiogram
ROS	Reactive oxygen species
NO	Nitric oxide
TC	Total cholesterol
TG	Triglycerides
LDL	Low‐density lipoprotein
BDNF	Brain‐derived neurotrophic factor
BACE1	mRNA level of β secretase
CREB	cAMP‐response‐element binding protein
ROCK‐1	Rho‐associated coiled‐coil containing protein kinase 1
GSH	Glutathione
GPx	Glutathione peroxidase
MDA	Malondialdehyde
pCREB	Phosphorylated cAMP response element‐binding protein
SIRT3	Mitochondrial sirtuin‐3
LDH	Lactate dehydrogenase
MMP	Mitochondrial membrane potential
TrkB	Tropomyosin receptor kinase B
NOX4	NADPH oxidase 4
GLUT4	Glucose transporter type 4
IR	Insulin resistance
AGEs	Advanced glycation end products
AMPK	Adenosine 5′‐monophosphate‐activated protein kinase
PI3K	Phosphatidyl inositol 3‐kinase
HO‐1	Heme oxygenase 1
Drp1	Dynamin‐related protein 1
PDGFs	Platelet‐derived growth factors
DAMPs	Damage‐associated molecular patterns
ECM	Extracellular matrix

## Author Contributions

**Muhammad Usman Khalid:** investigation (equal), validation (equal), writing – original draft (equal). **Muhammad Tauseef Sultan:** conceptualization (equal), supervision (equal). **Muhammad Maaz:** investigation (equal). **Shehnshah Zafar:** writing – original draft (equal). **Anum Shoukat:** data curation (equal). **Nudrat Khursheed:** writing – review and editing (equal). **Matteo Bordiga:** writing – review and editing (equal). **Amna Junaid:** writing – review and editing (equal).

## Conflicts of Interest

The authors declare no conflicts of interest.

## Data Availability

The authors have nothing to report.
